# The Action of the Voluntary Muscles

**Published:** 1862-05

**Authors:** Louis Mackall


ART. V.—*The Action of the Voluntary Muscles; by* LOUIS MACKALL, M. D. *(Extract from an unpublished work.)*

69. The action of the Voluntary Muscles is regulated by the law of nature that we have already stated, (37.) By virtue of this law, the determination of the nerve-fluid to a muscle is immediately followed by the active elongation of its fibres; the contraction of a muscle or of its fibres as immediately follows the withdrawal of the nerve-fluid. In other words, the presence of the nerve-fluid, its cause of action is, by virtue of this law, always attended by an active elongation of the fibres of a muscle; while the absence of a due portion of this fluid is as constantly attended by the contraction of its fibres.

By the aid of this law of nature, we have explained, I think, in a plain, intelligible and rational manner, the phenomena presented in the action of the involuntary muscles, that are placed about the walls of the tubes or hollow organs; by the same aid, the phenomena presented in the action of the voluntary muscles may be as rationally and intelligibly explained.

70. The minds of Physiologists of the present day are so fully impressed with the erroneous belief, so long inculcated in the books, that the active state of a muscle is that of contraction, and that the elongation of its fibres, (a state of which they possess a very imperfect and false notion,) which they are pleased to call relaxation, is a passive state, that the converse of this proposition, as presented above, will be at first regarded by them with prejudice and aversion. The true law of nature in relation to this subject, however, when its application is attended to, in the extensive application of it we propose to present, must in time be acknowledged; if truth, as is said, has a tendency to prevail over error in the human mind.

71. When we use the term muscle, it should be understood to embrace both the tendinous and fleshy portions of the organ; the same fibres, we conceive, extend through the whole, and are influenced by the cause of action, the nerve-fluid, as well in the tendon as in the fleshy part. The latter portion differs from the former probably only in the circumstance of being more largely supplied with nerves and blood-vessels, by means of which, when the muscle is in a state of action, the special circulation of the nerve-fluid (43) is established.

72. For the better illustration of their action, it will be found convenient to separate the voluntary muscles into the four following classes, viz: 1st. Such as are attached by one extremity only, having the rest of the muscle free to be extended or retracted at will; 2d. Such as are attached at both extremities, but are capable only of the same motion as the former class, that of direct extension or retraction; 3d. Such as are arranged circularly about the openings of the organs, and serve as sphincters; and 4th. Such as are attached to the bones at both extremities, and act by making use of the bones as levers.

*The Action of the First Class of Voluntary Muscles, that are attached by one extremity, and have the rest of the muscle free to be extended or retracted.*—73. Of the action of this class we will advert to that of the muscles of the tentacles in the lower orders of animals; and among the higher orders, to that of the muscles of the tongue, of the ciliary processes, and of the male organ of generation.

Perhaps there is no other instance in nature, in which is presented so fairly and clearly, the true condition of a muscle when in a state of action, as in that of the action of the muscles of the organ last mentioned.—Physiologists, by a resort to sophistry, of which we shall speak again, have ignored the action of these muscles, which is the same with that of all other muscles; for the state of erection produced by the action of these muscles is common to all the muscles of the living body when in action. Every muscle when in action is in a state of vital erection; which term should be understood to express simply the active elongation of the fibres, with a due supply of blood and of nerve-fluid to maintain this state. In the human subject, the true character of the organ of which we are speaking is masked, as it were, by the great development of the blood-vessels and nerves with which it is supplied; but this is not the case in some other species of animals, as in the horse, the ox, the hog, and the sheep. In these the main body of the organ is composed of longitudinal muscular fibres that may be actively elongated to a length of from twelve to twenty inches. The view of muscular action heretofore presented may be repeated in connection with the instance before us.

74. The suggestive impressions, appointed to precede the active state of this organ, having been duly received, the animal determines the nerve-fluid, in an extra supply, to its fibres through the sensory and motory nerves of this organ. In consequence, the proper longitudinal and other fibres of the organ become actively elongated, together with the fibres about the walls of the vessels and sinuses, in which there is an increased flow and accumulation of blood. With this accumulation of blood there is established the special circulation of the nerve-fluid to this point; the supply of this fluid is thus further enlarged, and the action of all the fibres is exalted to its extreme limit. This latter state of the organ is expressed by the term "venereal orgasm."

75. In this representation of the action of muscles, there are three points to which we desire to direct attention: the first is, the propriety of regarding the organ in question as a muscle attached at one extremity by two heads to the ossa pubis and ischia; the second is, *the active elongation* of the proper fibres of this muscle; and the third is, the *stffening or rigidity* of the same fibres when in action. The two latter points are particularly worthy of attention, as they occur in every instance of muscular action, and, what is very strange. have been entirely overlooked by physiologists.

76. The Ciliary processes are the small muscles or bundles of muscular fibres that are attached by one extremity to the margin or verge of the opening into the eye, called the pupil; and are so arranged that by their extension the pupil is diminished or the opening narrowed, and by their retraction the pupil is enlarged or the opening widened. Light is appointed to be the appropriate suggestive impression to precede the action of these muscles. When the eye, in its normal condition in the living body, is withdrawn from the light, the mind, not having received this suggestive impression, does not call into action these muscles, and they are consequently retracted, and the pupil is enlarged; but when the eye is brought into the light and the impression is made, the mind determines the nerve-fluid to these muscles, and they become extended in proportion to the supply of this fluid; by their combined action the pupil is diminished or narrowed, so as to exclude such quantity of light as might be injurious to the optic nerve.

Here again we have a vital erection, in the essential conditions—the active elongation, and apparently the stiffening of the fibres; and it is worthy of remark here, that in inflammation of these and the adjoining parts, as in iritis, there is established the special circulation of the nerve-fluid to these fibres, and they become persistently elongated, and the pupil continues to be narrowed even when the eye is withdrawn from the light.

77. The longitudinal muscular fibres of the tongue (with a view to the elucidation of the action of this organ) may all be regarded as one muscle, that is attached at one extremity, with the other parts free to be extended or retracted at will. The active elongation or extension of these fibres when innervated, or when in action is manifest in our own persons, if we confine our attention to the fibres in question; but this active elonga tion of the tongue in some of the lower orders of animals is very remarkable. Every one is familiar with the protrusion and elongation of the tongue of some of the domestic animals, as the dog, the cow, &c.

This action of the tongue of the serpent is deserving of particular notice. When the animal is aroused or excited, it erects its head and thrusts out of its mouth its forked tongue, t o the extent of several inches. The suddenness and celerity of the moti on of the organ forcibly reminds us of the appearance of a flash of lightning.

It is impossible, I believe, to suggest any rational explanation of this phenomenon, other than that of the innervation and consequent elongation of the fibres of the muscles that belong to this organ. The suddenness and celerity of the motion precludes all other agency but that of the nerve-fluid, passing along the nerves, which alone resembles in its motion that of the electric fluid.

The action of the tongue of the frog or toad, when it seizes its prey, is similar to that of the serpent.

78. A notable action of this kind is presented in that of the tongue of the chameleon. To facilitate its description, this organ may be divided into four parts; first, the anterior bulbous portion, formed by the interweaving of cellular and muscular tissues—with the appearance of the end of the trunk of the elephant, it is furnished like this with a fleshy forceps at the extremity, with which the animal seizes its prey; second, the middle or interior portion, *composed entirely of cellular tissue formed into a number of bands or hoops, having the intervals between them supplied with very loose and extensile meshes of this tissue;* third, the lingual bone, being a process from the centre of the os hyoides, arranged in the direction of the tongue, on which, when the tongue is retracted, the cellular bands and meshes of the second portion, and a part of the bulbous portion, which is hollowed out for this purpose, are stretched or drawn, like the finger of a glove drawn over the finger; and fourth, two well-developed muscles, having their fibres arranged longitudinally, and largely supplied with nerves and blood-vessels. These muscles arise from the cornua of the os hyoides, one from each, and being arranged one on each side of the tongue, or of the central cellular portion, are inserted in the bulbous extremity.

When in a state of repose, or when retracted within the mouth, the organ is from an inch to an inch and a half long; but when about to seize its prey, commonly an insect, the chameleon creeps to within seven or eight inches of the object, and then fixing its body, and taking aim, as it were, suddenly, and with the celerity of lightning, thrusts forward its tongue, and seizes the insect with the fleshy forceps at its extremity. The food thus taken is then drawn into the mouth by the retraction of the organ.

[TO BE CONTINUED.]

